# Reply to Spitz et al. Conclusions Are Not Supported by the Published Statistical Analysis. Comment on “López-Toledo et al. Flaxseed Improves Glucose and Lipid Metabolism in Mexican Subjects with Type 2 Diabetes: A Parallel Randomized Clinical Trial. *Nutrients* 2025, *17*, 709”

**DOI:** 10.3390/nu18010171

**Published:** 2026-01-05

**Authors:** Sabina López-Toledo

**Affiliations:** Center for Studies in Health Sciences and Disease, “Benito Juarez” Autonomous University of Oaxaca, Oaxaca 68000, Mexico; sabina.ltoledo@gmail.com

## 1. Correction of Figure 3

We sincerely thank the Editor and the commenting authors for their careful review and constructive observations regarding our article [1]. Below we address their concerns and provide updated analyses.

**Figure 3:**
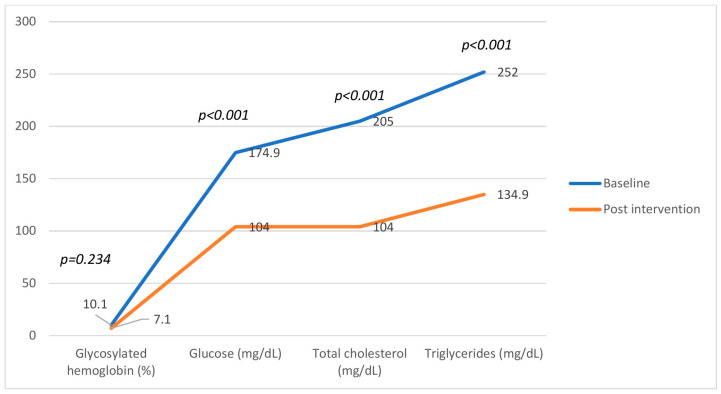
Behavior of biochemical parameters of participants in the intervention group (n = 82) before and after 12 weeks of flaxseed supplementation. Significant reductions were observed in fasting glucose, total cholesterol, and triglycerides (*p* < 0.001), while the change in glycated hemoglobin was not significant (*p* = 0.234). Data are presented as mean ± SD.

During manuscript preparation, Figure 3 (Behavior of biochemical parameters of participants in the intervention group) was inadvertently duplicated from Figure 2, leading to misrepresentation of the control group data. We regret this oversight. The textual results and underlying dataset remain correct and unaffected. The corrected Figure 3 and data are provided as follows.

## 2. Reanalysis to Avoid DINS Error

To address the concern about Difference in Nominal Significance (DINS) and to properly test between-group differences, we reanalyzed our data using analysis of covariance (ANCOVA) with baseline values as covariates. The results strongly confirm our original conclusions:

- Glycated Hemoglobin (HbA1c): F(1163) = 192.89, *p* < 0.001. Adjusted means (±SE): 7.15 ± 0.13% (intervention) vs. 9.75 ± 0.13% (control); adjusted difference: −2.60% (95% CI: −2.97 to −2.23).
- Glucose: F(1163) = 58.42, *p* < 0.001. Adjusted means (±SE): 100.9 ± 3.6 mg/dL vs. 139.9 ± 3.6 mg/dL; difference: −38.96 mg/dL (95% CI: −49.02 to −28.89).
- Total Cholesterol: F(1163) = 266.99, *p* < 0.001. Adjusted means (±SE): 144.3 ± 2.7 mg/dL vs. 207.8 ± 2.7 mg/dL; difference: −63.53 mg/dL (95% CI: −71.20 to −55.85).
- Triglycerides: F(1163) = 296.11, *p* < 0.001. Adjusted means (±SE): 130.8 ± 4.3 mg/dL vs. 235.8 ± 4.3 mg/dL; difference: −105.08 mg/dL (95% CI: −117.14 to −93.02).

## 3. Data Availability

De-identified raw data and the R/SPSS code used for these analyses have been deposited in Zenodo for independent verification [2].

## 4. Conclusions

The corrected figure and new ANCOVA analyses confirm that daily supplementation with 16 g of flaxseed for 12 weeks significantly improves glucose and lipid metabolism in adults with type 2 diabetes compared to the control. We respectfully request that the journal publish this Author’s Reply and the associated corrigendum to update the record.

Thank you for the opportunity to clarify and strengthen our findings.
